# Triarylborane Catalyzed Carbene Transfer Reactions Using Diazo Precursors

**DOI:** 10.1021/acscatal.1c04746

**Published:** 2021-12-17

**Authors:** Ayan Dasgupta, Emma Richards, Rebecca L. Melen

**Affiliations:** Cardiff Catalysis Institute, School of Chemistry, Cardiff University, Main Building, Park Place, Cardiff CF10 3AT, Cymru/Wales, United Kingdom

**Keywords:** diazoester, triarylborane, carbene transfer, diazo activation, catalysis

## Abstract

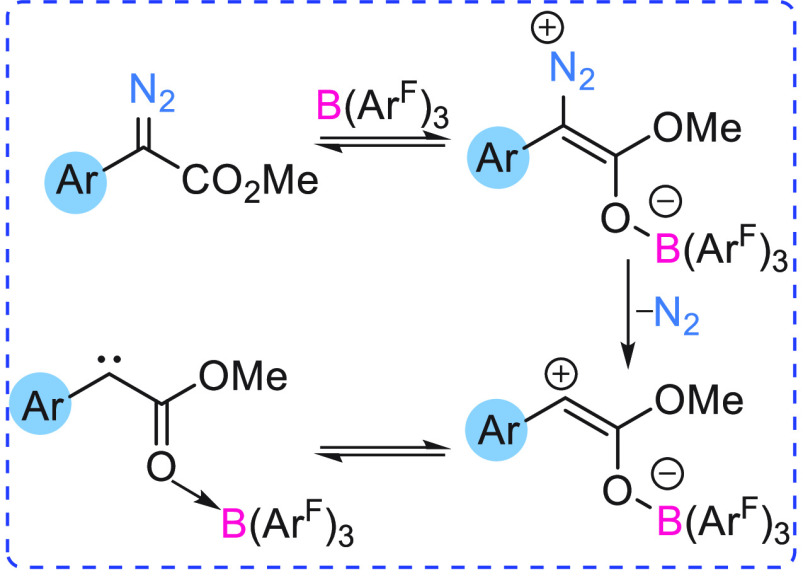

Reactive carbenes generated from diazo compounds are key intermediates for a range of organic reactions to afford synthetically useful organic compounds. The majority of these reactions have been carried out using transition metal catalysts. However, the formation of carbene intermediates using main group elements has not been widely investigated for synthetic purposes. Recent studies have demonstrated that triarylboranes can be used for the *in situ* generation of reactive carbene intermediates in both stoichiometric and catalytic reactions. These new reactivities of triarylboranes have gained significant attention in synthetic chemistry particularly in catalytic studies. The range of organic compounds that have been synthesized through these reactions are important as pharmaceuticals or agrochemicals. In this perspective, we highlight the recent progress and ongoing challenges of carbene transfer reactions generated from their corresponding diazo precursors using triarylboranes as catalysts. We also highlight the stoichiometric use of triarylboranes in which the boranes not only activate the diazo functionality to afford a carbene intermediate but also actively participate in the reactions as a reagent. The different mechanisms for activation and carbene transfer are described along with the mechanistic and computational studies that have aided the elucidation of these reaction pathways. Potential opportunities for the use of boranes as a catalyst toward different carbene transfer reactions and their future prospects are discussed.

## Introduction

Carbene transfer reactions using transition metal catalysts are well developed, and the conventional method to generate metal carbenoid species is from the reaction between diazo compounds and catalytic amounts of a transition metal.^1,2^ Transition metals including Fe, Cu, Pd, Pt, Ni, Rh, Ru, Pd, Ag, and Au are commonly employed as catalysts for the decomposition of diazo compounds to afford corresponding metal carbenoid species. In general, the metal-carbenoid intermediates are reactive and therefore readily can undergo a range of organic reactions.^3^ However, due to the high reactivity of the metal carbenoid species, occasionally lower selectivity is observed in stereoselective reactions.^4^ In recent years, borane mediated activation of nitrogen containing compounds has become a popular area of research following the groundbreaking work from Braunschweig which demonstrated that borylene species can effectively bind and activate dinitrogen.^5^ In particular, research groups have explored the activation of diazo compounds using triarylboranes. By employing a borane catalyst to generate the carbene intermediate, a range of highly regio- and stereoselective organic reactions have been successful. While metals have the ability to undergo synergic bonding and back-bonding interactions with the carbene intermediates, this is not the case for triarylboranes in which the central boron center behaves purely as an acceptor into the vacant *p*-orbital.^6^

Using fluorinated triarylboranes [B(Ar^F^)_3_], a range of carbene transfer reactions have been achieved including O–H/N–H/C–H insertion,^7^ azide transfer,^8^ carbonate transfer,^9^ C=C bond formation,^10^ carbocycle formation (cyclopropanation/cyclopropenation),^7c,11^ and the ring opening of heterocyclic compounds.^7c^ In this perspective, we will discuss the activation of diazo compounds toward N_2_ release followed by the subsequent reactions of the carbene-borane bound intermediate in both stoichiometric and catalytic reactions.

## Activation of Diazo Compounds Using Stoichiometric B(Ar^F^)_3_

Although diazo activation using a metal catalyst has been widely studied, the mode of activation of diazo compounds using boranes has not been examined extensively. Recently, the activation of diazo compounds has been investigated both experimentally and computationally using stoichiometric and catalytic boranes. Most of the studies to date have focused on the more stable α-aryl α-diazoesters.

**Scheme 1:**
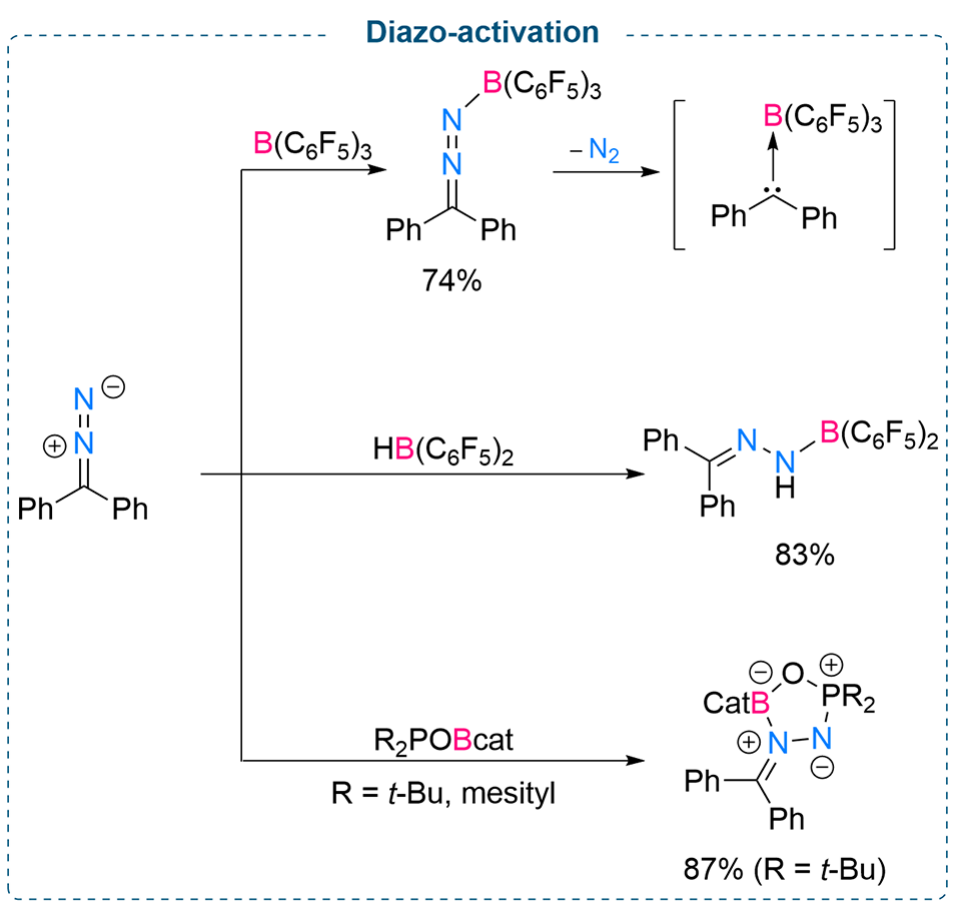
Activation of Diphenyldiazomethane Using Stoichiometric B(C_6_F_5_)_3_ (Top), HB(C_6_F_5_)_2_ (Middle), and an FLP (Bottom)

However, in 2017, Stephan and co-workers demonstrated that B(C_6_F_5_)_3_ can activate diphenydiazomethane as Lewis acidic boranes readily interact with the nitrogen functionality of diazo compounds, leading to the formation of Lewis acid–base adducts.^12^ The stoichiometric reaction between Ph_2_CN_2_ and B(C_6_F_5_)_3_ at low temperature (−78 °C) afforded the proposed N_diazomethane_ → B adduct Ph_2_CN_2_B(C_6_F_5_)_3_. Formation of this adduct was confirmed by multinuclear NMR spectroscopy (^11^B and ^19^F). The authors proposed that rapid evolution of N_2_ at elevated temperature from Ph_2_CN_2_B(C_6_F_5_)_3_ leads to the formation of a new C_carbene_ → B adduct Ph_2_C(B(C_6_F_5_)_3_) (Scheme 1, top). DFT calculations showed loss of N_2_ from the adduct Ph_2_CN_2_B(C_6_F_5_)_3_ to form C_carbene_ → B is exergonic by about 53 kcal/mol. Furthermore, formation of the C_carbene_ → B adduct was also supported by the bond length of C_carbene_ → B (1.66 Å) which was found to be comparable with the C–B bond length in B(C_6_F_5_)_3_. Piers’ borane [HB(C_6_F_5_)_2_] also readily reacts with diphenyldiazomethane to afford a stable compound (Scheme 1, middle). It is noteworthy to mention that this is the first example of formation and 1,1-hydroboration of a N–N bond. Moreover, further studies by Stephan and co-workers revealed that the reactivity pattern between Ph_2_CN_2_ and B(C_6_F_5_)_3_ changed in the presence of Cp*_2_Co. A stoichiometric reaction between Ph_2_CN_2_, B(C_6_F_5_)_3_, and Cp*_2_Co afforded a mixture of [Cp*_2_Co][Ph_2_CNNHB(C_6_F_5_)_3_ and [Cp*Co(C_5_Me_4_CH_2_B(C_6_F_5_)_3_)].

A single electron transfer from Co^II^ to the diazomethane-borane adduct Ph_2_CN_2_B(C_6_F_5_)_3_ was proposed to account for the formation of [Cp*Co(C_5_Me_4_CH_2_B(C_6_F_5_)_3_)].^13^ Furthermore, in 2020, Stephan and co-workers disclosed that a stoichiometric reaction between Ph_2_CN_2_ and the oxygen-linked geminal FLP complexes R_2_POBcat (R = *t*-Bu, mesityl) (derived from the reaction between phosphine oxides *t-*Bu_2_P(O)H/Mes_2_P(O)H and ClBcat) afforded Ph_2_C(N_2_)BcatOPR_2_.^14^ Formation of these BOPN_2_ five-membered heterocyclic compounds was confirmed by single crystal X-ray diffraction. Although the authors were able to isolate Ph_2_C(N_2_)BcatOP*t*-Bu_2_ in 87% yield, isolation of Ph_2_C(N_2_)BcatOPMes_2_ as a pure compound failed (Scheme 1, bottom).

As evident from the above discussions, triarylboranes can be employed to generate carbene species. We and others have also observed that Lewis acidic triarylboranes can also actively participate in organic reactions when employed in stoichiometric amounts. In 2012, Stephan and co-workers disclosed an interesting synthetic protocol where insertion of diazomethane into one or two B–C bonds of electrophilic pentafluoroarylboranes was demonstrated. The authors examined the outcome of the stoichiometric reactions between Lewis acidic boranes such as B(C_6_F_5_)_3_, PhB(C_6_F_5_)_2_, and ClB(C_6_F_5_)_2_ and various diazomethanes bearing trimethylsilane (TMS), diphenyl, and pentafluorophenyl substituents. A range of new sterically encumbered electrophilic borane derivatives were synthesized in good to excellent yields (60–97%).^15^

**Scheme 2:**
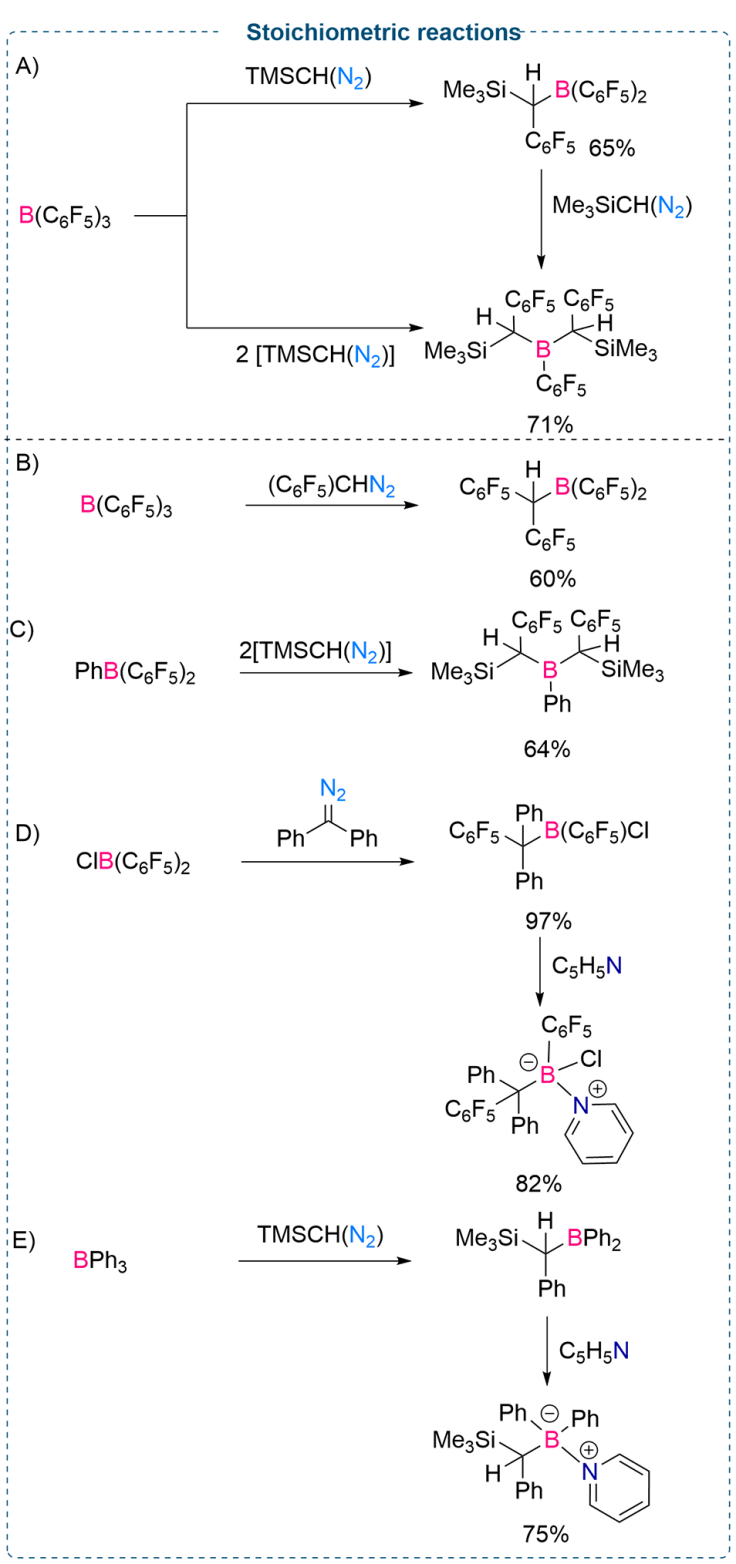
Stoichiometric Reaction between B(Ar^F^)_3_ and Diazo Compounds

A stoichiometric amount of B(C_6_F_5_)_3_ in CH_2_Cl_2_ was reacted with TMSCH(N_2_) (2.0 M solution in hexane) at −78 °C to afford the new borane (TMSCH(C_6_F_5_))B(C_6_F_5_)_2_ in 65% yield (Scheme 2A) formed from the insertion of the carbene into a B–C_6_F_5_ bond. Moreover, the reaction between B(C_6_F_5_)_3_ and 2 equiv of TMSCH(N_2_) at −78 °C afforded the product (TMSCH(C_6_F_5_))_2_B(C_6_F_5_) in 71% yield (Scheme 2A), formed from the double insertion of the diazomethane into two B–C bonds of B(C_6_F_5_)_3_. Similarly, B(C_6_F_5_)_3_ reacts with (C_6_F_5_)CHN_2_ stoichiometrically to afford (C_6_F_5_)_2_CHB(C_6_F_5_)_2_ in 60% yield (Scheme 2B). Furthermore, stoichiometric reactions between PhB(C_6_F_5_)_2_ and TMSCH(N_2_) were investigated. Double insertions of TMSCH(N_2_) into B–C bonds led to the formation of (TMSCH(C_6_F_5_))_2_B(C_6_F_5_) in 64% yield (Scheme 2C).

The authors observed that although the reaction between TMSCH(N_2_) with ClB(C_6_F_5_)_2_ afforded a complex mixture of different products, the reaction of Ph_2_C(N_2_) and ClB(C_6_F_5_)_2_ exclusively afforded (C_6_F_5_)ClB(CPh_2_(C_6_F_5_)) in 97% yield (Scheme 2D).

This electrophilic borane can be further treated with weakly coordinating nucleophilic pyridine to form a salt ((C_6_F_5_)(Cl)(CPh_2_(C_6_F_5_))B(py)) (Py = pyridine) in a good yield (82%). Likewise, the reaction between BPh_3_ and TMSCH(N_2_) led to the formation of (TMSCH(C_6_H_5_)BPh_2_) which can further react with pyridine to afford TMSCH(C_6_H_5_)B(Py)(C_6_H_5_)_2_)Ph_2_ in 75% yield (Scheme 2E).^16^

**Scheme 3:**
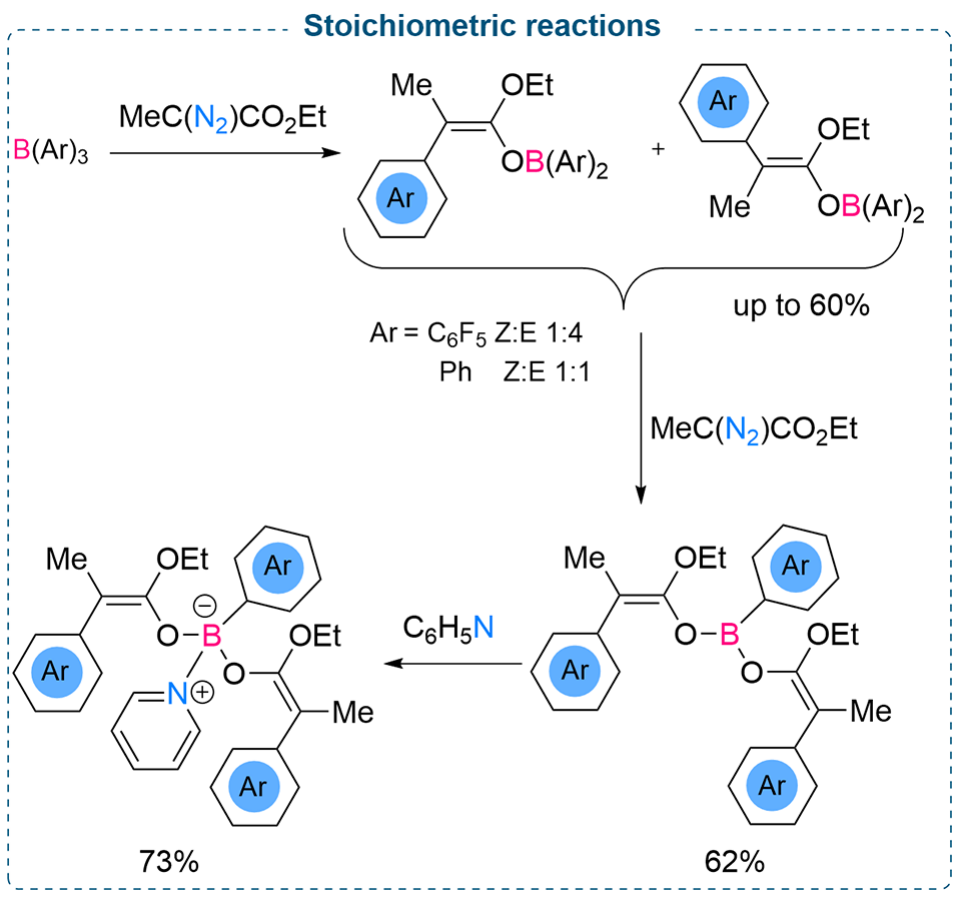
Stoichiometric Reaction between B(Ar)_3_ and Ethyl α-Diazomethyl Acetate

Continuing the exploration of a single/double insertion reaction into the B–C bond of boranes, Stephan and co-workers observed that the stoichiometric reaction between ethyl α-diazomethyl acetate and B(C_6_F_5_)_3_ produced boron enolates from single or double insertion of the diazo compound into a B–C bond (Scheme 3).^21^ Further treatment of the enolate with pyridine led to the formation of the corresponding adduct in 73% yield. These methods demonstrated the derivatization of B–C, B–H, and B–Cl bonds employing stoichiometric reactions between the boranes and diazomethanes. This methodology shows the divergent ways to make a variety of new bulky, secondary, and tertiary substituted Lewis acidic boranes.

**Scheme 4:**
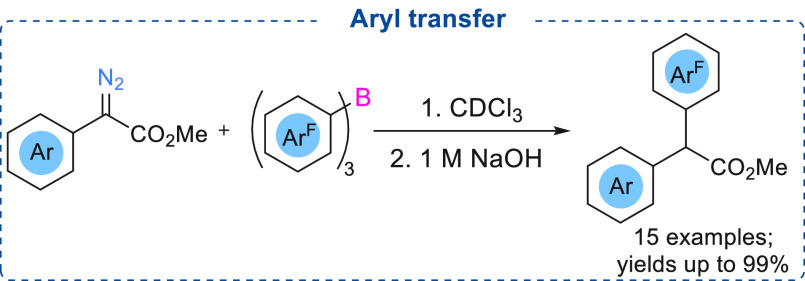
Stoichiometric Reaction between B(Ar^F^)_3_ and α-Aryl α-Diazoesters

In 2019, we disclosed the stoichiometric reaction between α-aryl α-diazoacetates and triarylboranes to afford synthetically useful 3,3-disubstituted benzofuranones bearing a quaternary carbon center.^17^ Our initial results reveal that a stoichiometric reaction between triarylboranes [BPh_3_, B(4-FC_6_H_4_)_3_, B(2,6-F_2_C_6_H_3_)_3_, B(C_6_F_5_)_3_, and B(3,4,5-F_3_C_6_H_2_)_3_] with different diazoesters led to the formation of an enolate product in which the aryl group from BAr_3_ has been transferred to the diazoester, similar to that reported by Stephan above (Scheme 3). It was noted that the yields of such reactions were improved with increasing Lewis acidity of the borane (BPh_3_ < B(4-FC_6_H_4_)_3_ < B(2,6-F_2_C_6_H_3_)_3_ < B(C_6_F_5_)_3_ < B(3,4,5-F_3_C_6_H_2_)_3_) with more Lewis acidic boranes able to transfer more aryl groups depending upon the substrates (Scheme 4).

**Scheme 5:**
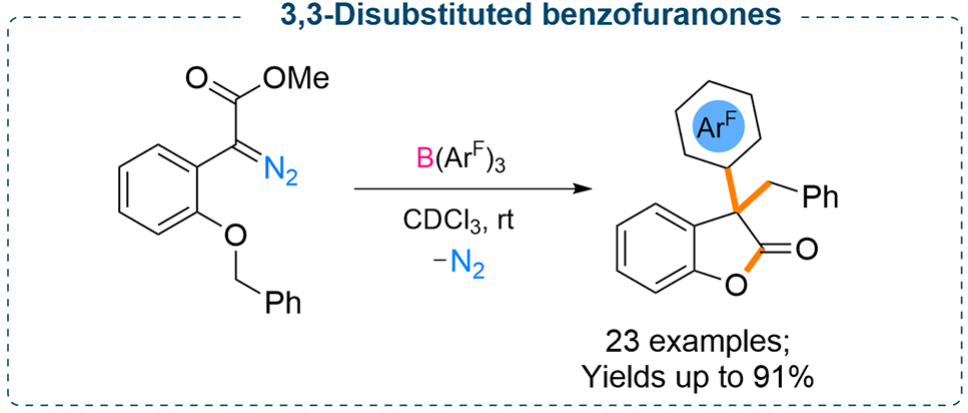
B(Ar^F^)_3_ Mediated Formation of Oxygen Heterocycles

**Scheme 6:**
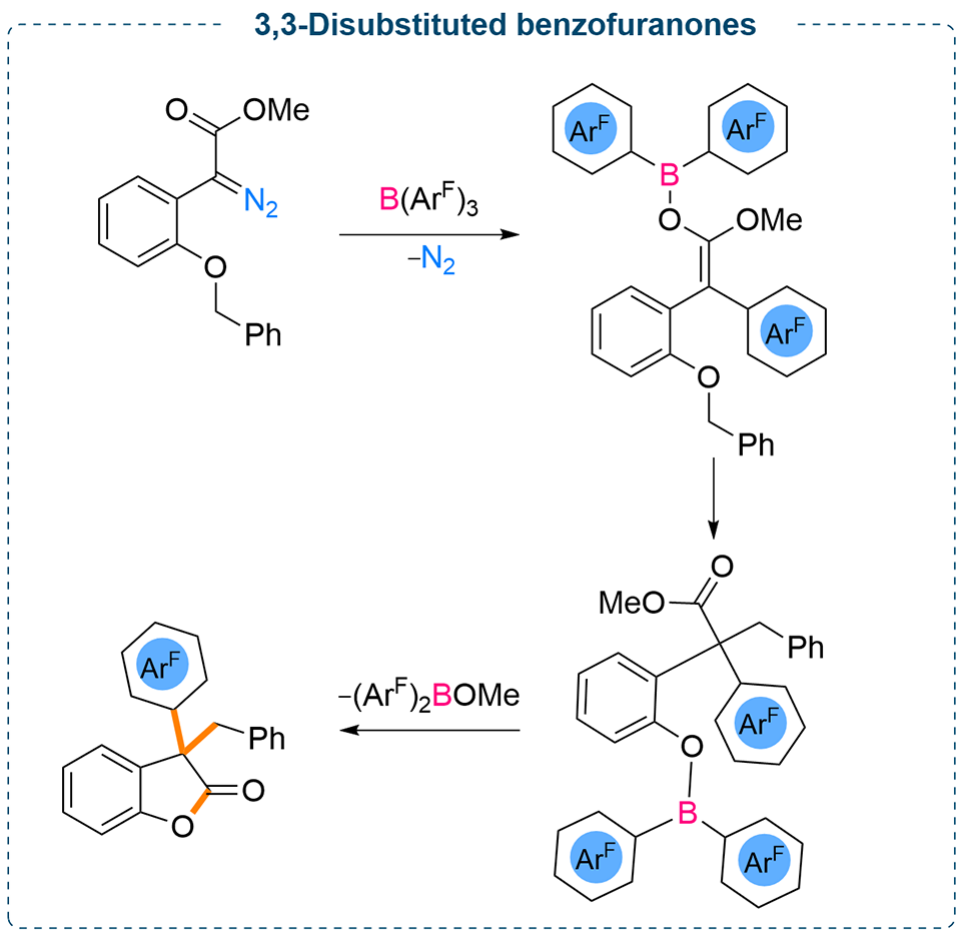
Formation of 3,3-Disubstituted Benzofuranones from the Stoichiometric Reaction between B(Ar^F^)_3_ and Diazoesters

Interestingly, when 2-benzyloxy substituted diazo derivatives were employed for such reactions, an unexpected attack of the generated boron enolate onto the benzyl group occurred followed by an aryl group transfer from the BAr_3_ which resulted in the formation of 3,3-disubstituted benzofuran-2-(3*H*)-ones in good to excellent yields (up to 91%) (Schemes 5 and 6). Moreover, changing the heteroatom from oxygen to sulfur/nitrogen also worked successfully, and the corresponding sulfur/nitrogen based five-membered heterocyclic compounds were obtained in moderate yields (up to 55%).

## Activation of Diazo Compounds Using Catalytic B(C_6_F_5_)_3_

**Figure 1:**
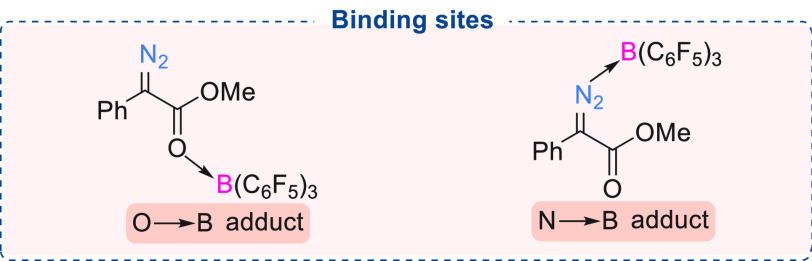
Possible binding modes of B(C_6_F_5_)_3_ to a diazoester.

**Scheme 7:**
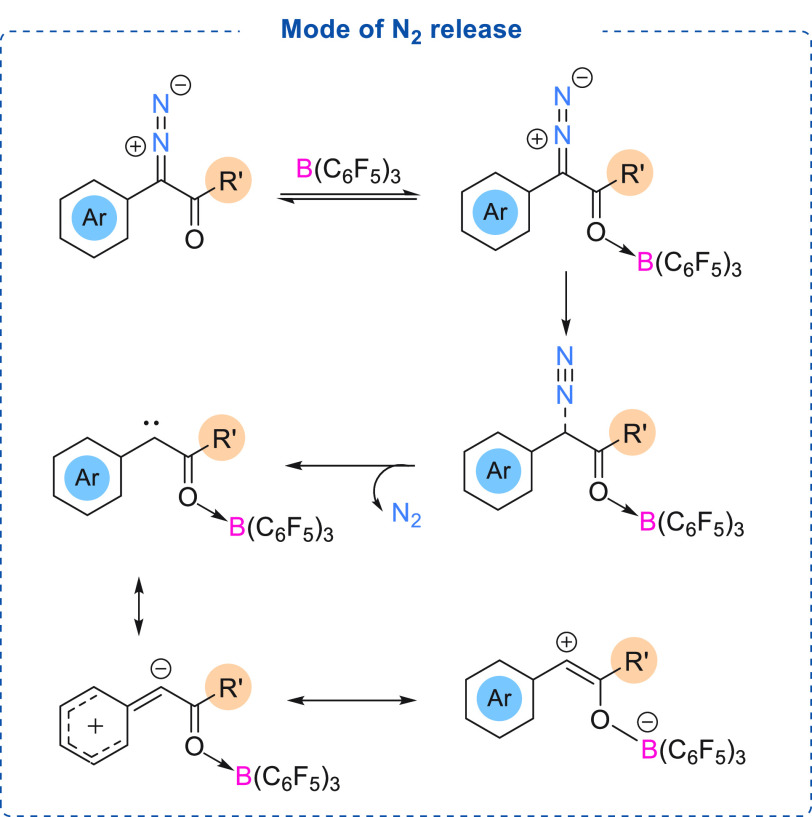
Diazo Activation Using Catalytic B(C_6_F_5_)_3_

We and others have investigated the factors affecting the ease of N_2_ release from α-aryl α-diazocarbonyl compounds. Although there are two possible coordination sites in an α-aryl α-diazoester (Figure 1), Lewis acidic boranes show preferential binding at the carbonyl functionality to form the O → B adduct which is energetically more favorable than the N → B adduct. B(C_6_F_5_)_3_ coordination makes N_2_ release more facile, as the C–N bond (in the O → B adduct) is weaker (1.334 Å) compared to the uncoordinated α-aryl α-diazoester (1.318 Å).^7c^ O → B adduct formation also causes a shortening of the C–C bond (1.470 Å to 1.436 Å). These factors result in more facile N_2_ release in the presence of the borane (Scheme 7). As calculated by DFT studies, the interaction between the Lewis acidic borane and α-aryl α-diazoester depends on the electronic nature of the substituent present on either the aryl ring or the carbonyl functionality of the α-aryl α-diazoester. Here, a strong correlation between the intrinsic stability of the formed carbene and the activation barrier for N_2_ release from the α-aryl α-diazocarbonyl was calculated.^18^ We observed that in the absence of any borane catalyst, the ease of N_2_ release is favored by electron donating substituent(s) (NMe_2_/NH_2_/OMe) attached at the *para* position of the aryl ring. Conversely, a highly electron withdrawing (NO_2_) group required higher activation energy. The activation free energy for N_2_ release, in the absence of a Lewis acid, was calculated to be between ∼26 kcal/mol and ∼35 kcal/mol. However, the influence of the substituents at the carbonyl functionality was found to have minimal effect. The lower activation energy toward N_2_ release from an α-aryl α-diazoester bearing an electron donating group (NMe_2_/NH_2_/OMe) attached at the *para* position of the aryl ring was attributed to the greater stability of the formed carbene due to the π-donation from the aromatic ring (Scheme 7). DFT studies demonstrate that the stability of the formed carbene species (O → B adduct) can be further enhanced (compared to the uncatalyzed process) in the presence of a Lewis acidic borane due to an increase in the contribution of this resonance structure (Scheme 7) which stabilizes the carbene. Therefore, in the presence of Lewis acidic B(C_6_F_5_)_3_, the ease of N_2_ release to afford the carbene species is kinetically and thermodynamically favored. As evidenced from the DFT calculations, the activation barrier (Δ*G*^‡^_2_) for N_2_ release for an α-aryl α-diazoester bearing a NMe_2_ functionality on the aryl ring (*para*-position), was found to be 15.4 kcal/mol, whereas in the absence of a Lewis acid catalyst, N_2_ release required an activation energy of 26.7 kcal/mol (Δ*G*^‡^_1_).

Further DFT studies demonstrated that the electronic nature of the substituent at the carbonyl functionality (R′, Scheme 7) could also affect carbene formation in the presence of B(C_6_F_5_)_3_. The ease of N_2_ release is favored by substituents lacking lone pairs of electrons.

**Scheme 8:**
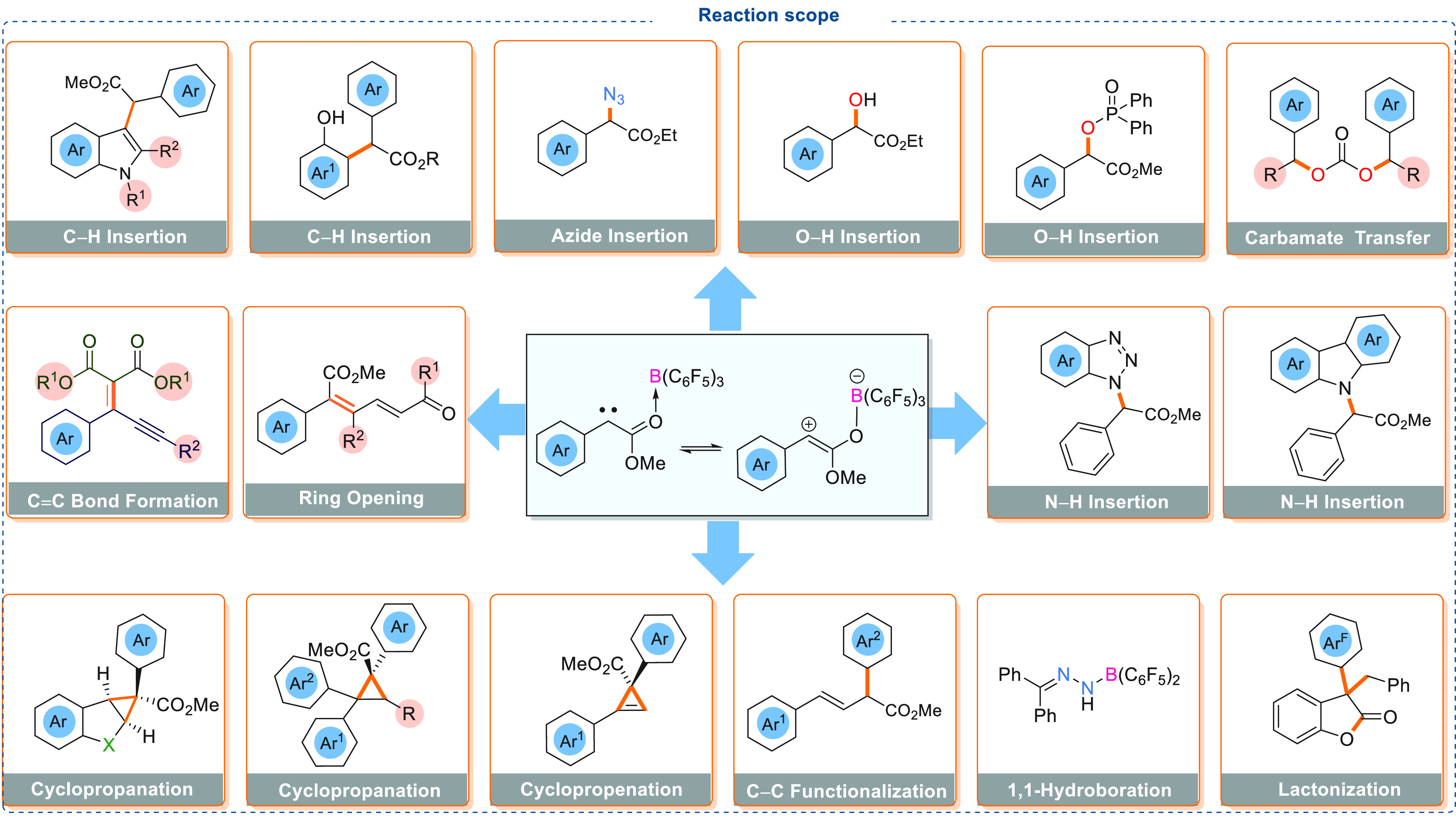
Generic Overview of B(C_6_F_5_)_3_ Activation of Diazo Compounds and Subsequent Reactivity

As is clear from the above discussions, triarylboranes are capable of activating the diazoester to generate a carbene intermediate which is electrophilic in nature due to the presence of the electron withdrawing triarylborane; therefore, the reactive carbene intermediate can readily react with a range of substrates to afford numerous useful products. Scheme 8 represents the divergent reactivity of the carbene intermediate using triarylboranes in stoichiometric and catalytic reactions. A summary of these recent developments will be discussed in the following sections.

## X–H (X = O, N, and C) Insertion

The resulting O → B adduct following N_2_ loss was found to be highly electrophilic^7c^ which has resulted in their use in a range of insertion reactions with nucleophiles including O–H, N–H, and C–H insertion.

**Scheme 9:**
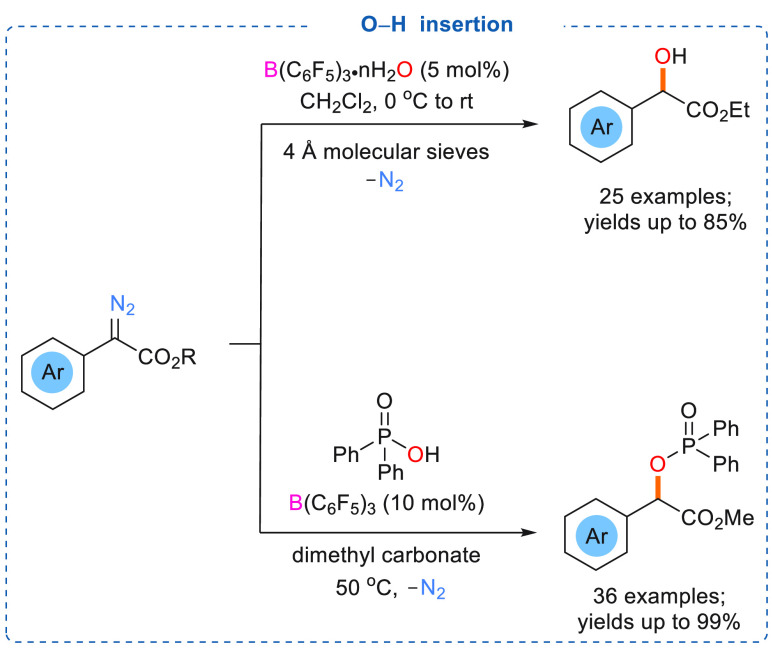
B(C_6_F_5_)_3_ Catalyzed O–H Insertion into α-Artyl α-Diazoesters (Top) and O–H Insertion of α-Aryl α-Diazoesters into Phosphinic Acids (Bottom)

**Scheme 10:**
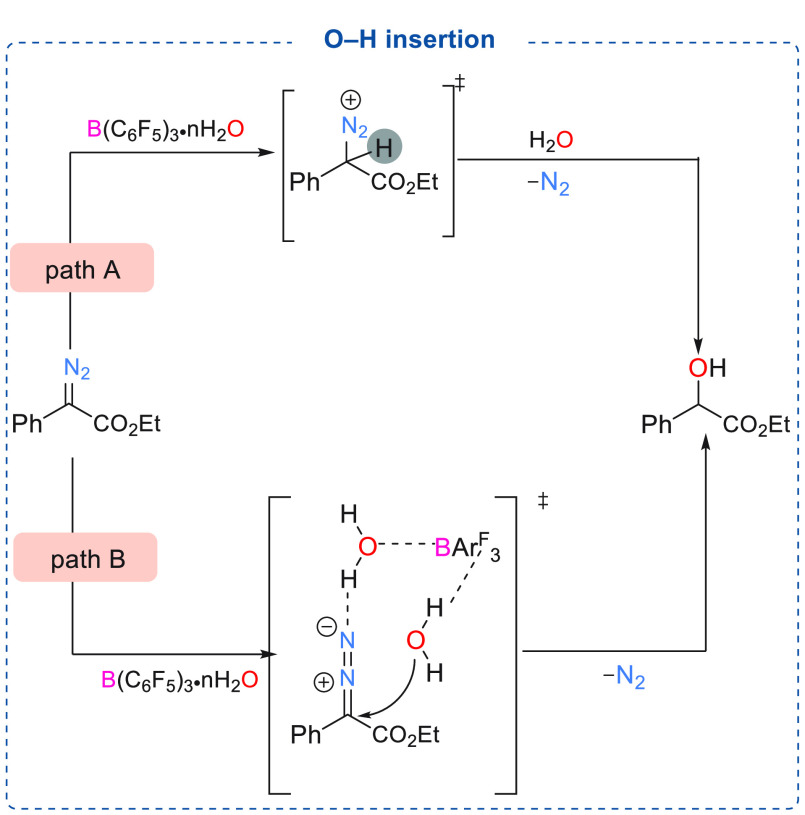
Mechanistic Details of B(C_6_F_5_)_3_ Catalyzed O–H Insertion into α-Aryl α-Diazoesters

In 2018, Tang and co-workers demonstrated O–H bond insertion reactions using α-aryl α-diazoesters with catalytic amounts of borane. However, Brønsted acidic B(C_6_F_5_)_3_·*n*H_2_O was used as a catalyst (Scheme 9, top) and water acted as the nucleophile to generate O–H bond inserted products (25 examples) in good to excellent yields (up to 85%).^7d^ Two possible mechanisms were suggested to account for the formation of such products. In the first mechanism, B(C_6_F_5_)_3_·*n*H_2_O acts as a proton source and donates a proton to the α-aryl α-diazoester which facilitates the water attack at the electrophilic carbon center to afford the desired products (Scheme 10, path A). Another possibility is that B(C_6_F_5_)_3_·*n*H_2_O acts as a bifunctional catalyst, which can activate the diazoester through protonation and promote subsequent nucleophilic attack by the water molecule leading to the formation of the OH inserted products.

A hydrogen bond network between B(C_6_F_5_)_3_, water, and N_2_ was used to explain the reaction mechanism (Scheme 10, path B).^7d^ Using a similar reaction strategy, Jiang and co-workers demonstrated O–H insertion into α-aryl α-diazoesters using phosphinic acids and catalytic B(C_6_F_5_)_3_ (Scheme 9, bottom).^19^ The reaction between nucleophilic phosphinic acids and different electrophilic α-aryl α-diazoesters afforded a range of α-phosphoryloxy carbonyl compounds in good to excellent yields (up to 99%).

Other types of insertion reactions including N–H insertion reactions have also been carried out using catalytic amounts of triarylboranes.

**Scheme 11:**
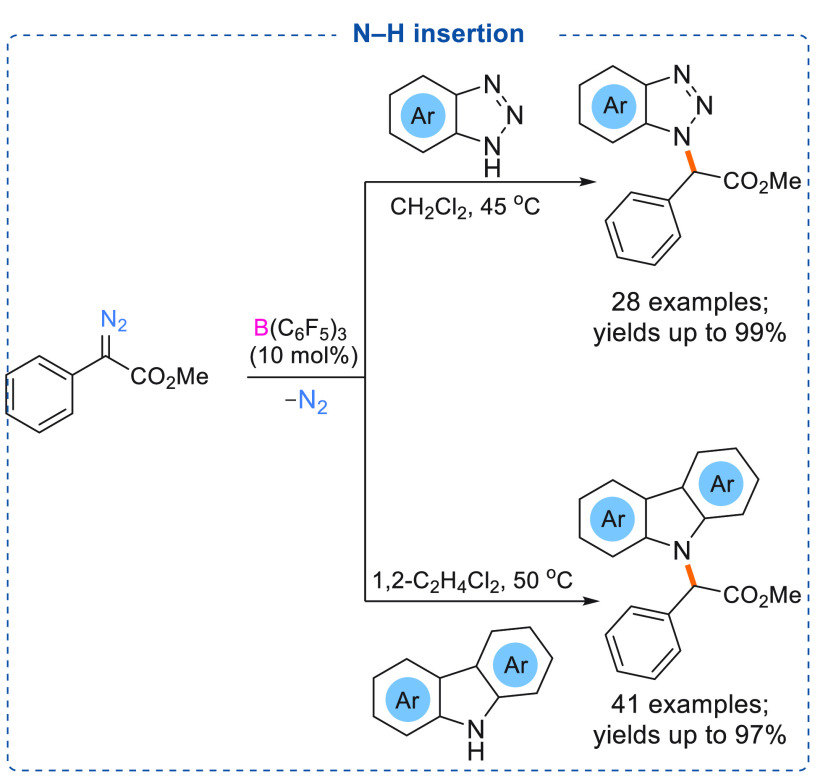
B(C_6_F_5_)_3_ Catalyzed N–H Insertion of Benzotriazole (Top) and Carbazole (Bottom) Using α-Aryl α-Diazoesters

Construction of a C–N bond without the use of precious transition metal catalysts is important as many nitrogen heterocycles are present in medicinal compounds, and trace amounts of toxic precious transition metal impurities in the target molecules are not acceptable. Therefore, the use of nontoxic triarylborane catalysts for C–N coupling reactions is highly beneficial.^20^ In 2021, Stephan revealed that a boron enolate (O → B adduct) (Figure 1) can readily react with the basic nitrogen center of weakly basic aromatic heterocyclic compounds to undergo N–H insertion reactions. Benzotriazoles were employed for a reaction in combination with α-aryl α-diazoesters to afford site-selective N1-alkylation of benzotriazoles in good to excellent yields (up to 99%, 28 examples) using catalytic B(C_6_F_5_)_3_ (Scheme 11, top).^7a^ Using very similar reaction conditions, Koenigs and co-workers have shown that unprotected carbazoles can undergo N–H insertion reactions with α-aryl α-diazoesters using 10 mol % B(C_6_F_5_)_3_. A wide substrate scope (41 examples) with near quantitative yields (up to 97%) of the *N*-alkylated products was reported (Scheme 11, bottom).^7b^

**Scheme 12:**
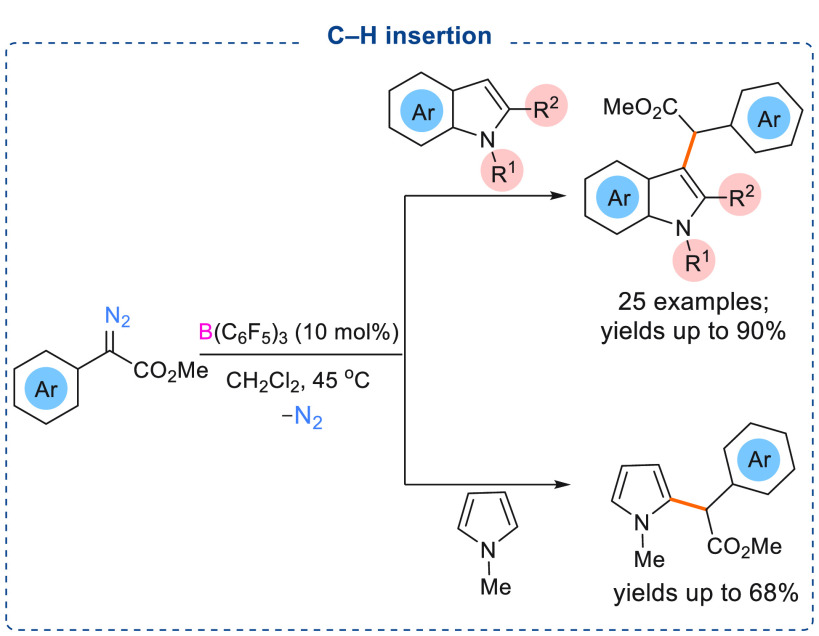
B(C_6_F_5_)_3_ Catalyzed C–H Insertion of Indoles and Pyrroles with α-Aryl α-Diazoesters

C–H bond functionalization is an important area of research in organic chemistry, and the development of facile metal-free synthetic methods toward C–H bond activation is an important topic. Metal-free carbene transfer into C–H bonds is a synthetically useful reaction in modern organic syntheses. As the interaction of a Lewis acidic borane with an α-aryl α-diazoester produces a highly electrophilic O → B adduct, carbene insertion into electron-rich C–H bonds would be an ideal way to afford C–H functionalized electron-rich heteroarenes. Recently, we have demonstrated that electron-rich *N*-heterocycles such as indoles and pyrroles can react with different α-aryl α-diazoesters to afford chemoselective C3 C–H inserted products in good to excellent yields (25 examples, yields up to 90%) (Scheme 12).^7c^ Surprisingly, when unprotected indoles were employed for the reaction with an α-aryl α-diazoester, the formation of N–H inserted products was not observed. Instead, unprotected indoles reacted with α-aryl α-diazoesters chemoselectively to afford C3 C–H inserted products in good yields (up to 70%).

Extensive DFT studies showed that the reaction between indoles and an α-aryl α-diazoester in the presence of catalytic B(C_6_F_5_)_3_ (10 mol %) initially afforded the kinetically controlled C2, C3 cyclopropane product, but the formed three-membered ring opened easily to generate a carbocation species (alpha to the N center). Finally, migration of the C3 hydrogen to the diazoester afforded the C3 C–H inserted product (Scheme 12, top).^7c^ A deuterium labeled study confirmed this C–H/D insertion. Using the optimized reaction conditions, pyrrole was found to undergo an electrophilic substitution at the C2 position (Scheme 12, bottom).

**Scheme 13:**
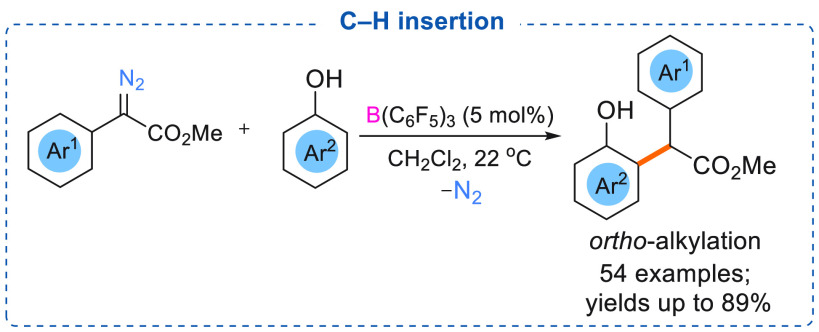
B(C_6_F_5_)_3_ Catalyzed *ortho*-Alkylation of Phenols

In 2016, Zhang and co-workers demonstrated that catalytic amounts of B(C_6_F_5_)_3_ (5 mol %) can be used for site selective substitution of unprotected phenols with α-aryl α-diazoesters (Scheme 13).^7e^ Reactions between various substituted phenols and α-aryl α-diazoesters were investigated, and *ortho*-C–H inserted products were obtained in moderate to good yields (54 examples, yields up to 89%). These reactions showed good chemoselectivity, and *para-*C–H inserted products were only isolated in small amounts. Mechanistic studies revealed that the high selectivity for the *ortho*-position was due to a hydrogen-bond directed process involving hydrogen bonding between a fluorine atom of B(C_6_F_5_)_3_ and the hydroxy group of the phenol. Moreover, the authors identified the hydroxy group as a proton source for these reactions, supported by a deuterium labeled study. To support the hydrogen-bond directed mechanism for the *ortho* alkylated products, anisole was employed for the reaction, and poor yields of the C–H inserted products (*ortho*/*para*) were obtained. In a similar study, an investigation by Koenigs demonstrated that electron-rich aromatic compounds can undergo C–H insertion (C-3 position) reactions with α-aryl α-diazoesters using B(C_6_F_5_)_3_ as a catalyst.^7b^

## Azide/Carbamate Insertion

Similar to O–H insertion into α-aryl α-diazoesters, strong nucleophiles such as azides can also be used for introducing nitrogen atoms into organic compounds using catalytic triarylboranes. As azides can participate in various organic reactions, including the synthesis of nitrogen heterocycles, a metal-free synthesis of organic azides would be very useful. In contrast with transition metal catalysts, triarylborane catalysts can, in some cases, demonstrate superior selectivity and degradation of azide products is minimized.

**Scheme 14:**
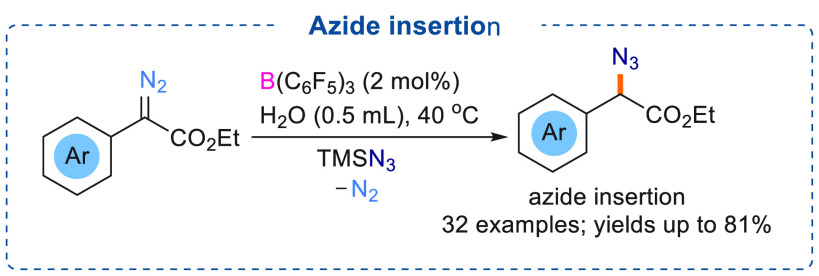
B(C_6_F_5_)_3_ Catalyzed Azide Insertion

Investigations by Tang demonstrated that nucleophilic azides can readily react with diazoesters and undergo azide insertion reactions using catalytic amounts of B(C_6_F_5_)_3_ (Scheme 14).^8^ Trimethylsilyl azide (TMSN_3_) was used as the azide source.

Mild reaction conditions were utilized for the selective α-azide α-carbonyl compound formation (32 examples) in good to excellent yields (up to 81%). These reactions showed a broad functional group tolerance. Employing a diazo substrate bearing alkene/alkyne functional groups for the azide insertion reaction showed no formation of carbocycles, and the alkene/alkyne site was untouched. For these reactions, the authors suggested the Lewis acidic boranes prefer to bind at the N_2_ functionality and TMSN_3_ was activated by carbonyl functionality of the diazoester. Subsequent nucleophilic attack of TMSN_3_ at the diazo compound eliminates N_2_ and leads to the formation of a silyl enol ether intermediate (mixture of *E*/*Z* isomers). Acid hydrolysis (silica gel chromatography) of the silyl enol ether finally afforded the azide inserted products.

**Scheme 15:**
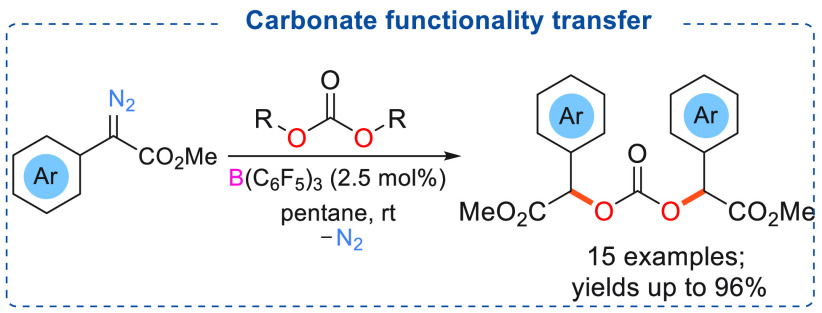
B(C_6_F_5_)_3_ Catalyzed Carbonate Functionality Transfer to α-Aryl α-Diazoesters

In 2019, Prabhu and co-workers also observed that an α-aryl α-diazoester generates the electrophilic carbene intermediate in the presence of catalytic amounts of B(C_6_F_5_)_3_ (2.5 mol %). This was found to readily react with di-*tert*-butyl dicarbonate (carbonate donor) to afford the desired products (Scheme 15). The electrophilic carbene center of the diazoester reacted with nucleophilic carbonates to afford the dialkylated carbonate compounds in good to excellent yields (up to 96%, 15 examples).^9^ It is presumed that the *tert*-butyl group is eliminated as isobutylene.

## Carbocycle Formation

Efficient and facile synthesis of small carbocycles using mild reaction conditions are significant as three-membered carbocycles are key motifs in biologically active compounds and are versatile synthetic intermediates in the synthesis of functionalized cycloalkanes.^21^ Transition metal catalyzed decomposition of diazoesters and subsequent reaction with olefins is the ubiquitous method to afford the three-membered carbocycle.^22^

However, analogous triarylborane catalyzed cyclopropanation is rather unusual. Recent studies demonstrated that catalytic amounts of triarylborane can catalyze the decomposition of a diazoester, and further reaction of the carbene intermediate with alkenes or alkynes can afford the cyclopropane or cyclopropene rings. As discussed earlier, due to high reactivities of metal carbenoid species, lower stereoselectivities are sometimes observed. Interestingly, the use of a triarylborane as the catalyst demonstrated very high diastereoselectivities in these reactions.

**Scheme 16:**
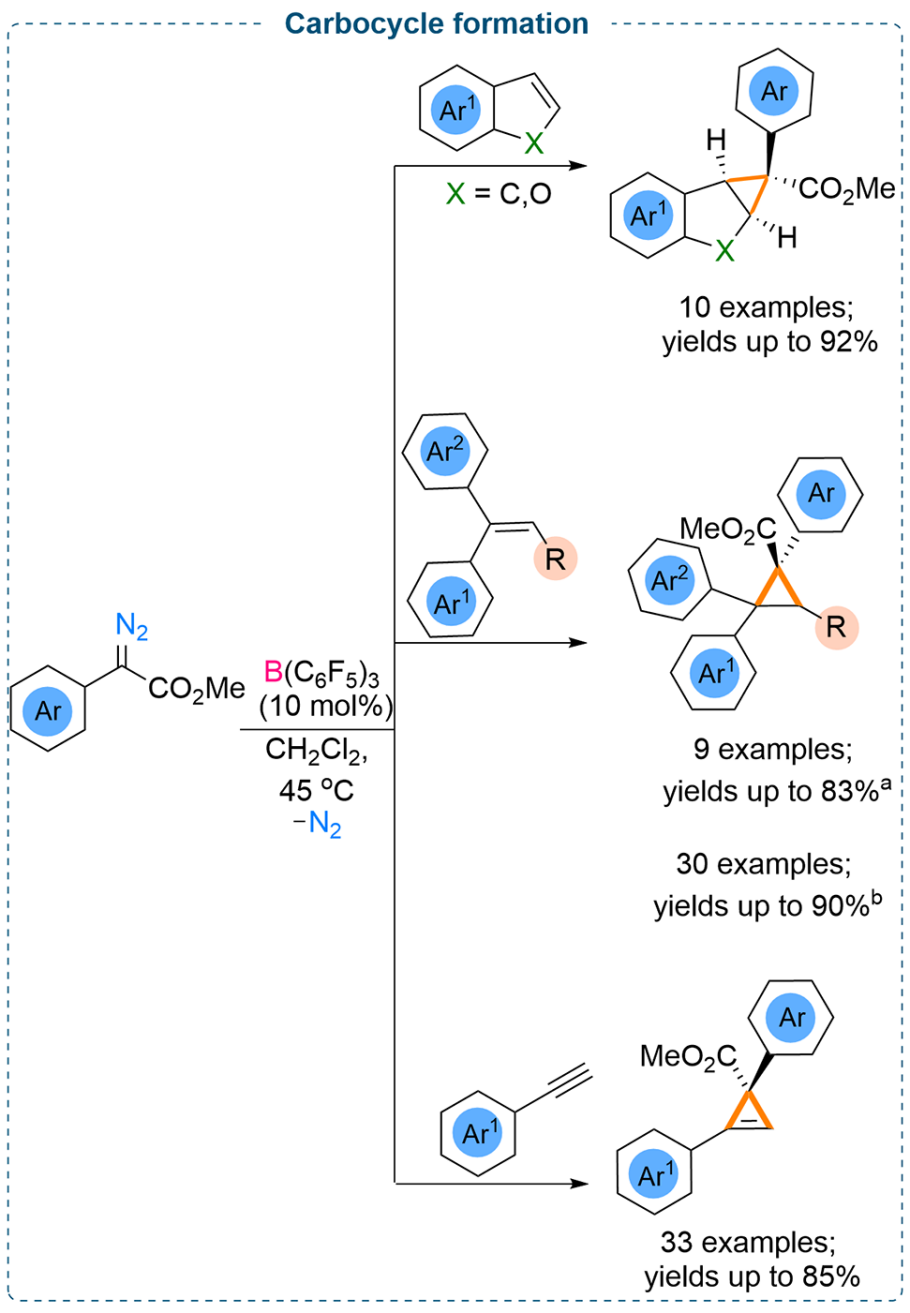
B(C_6_F_5_)_3_ Catalyzed Carbocycle Formation Using α-Aryl α-Diazoesters Melen, 2020. Wilkerson-Hill, 2020.

Our recent findings demonstrate that B(C_6_F_5_)_3_ can be employed as an efficient catalyst for the synthesis of three-membered carbocycles. Decomposition of donor–acceptor α-aryl α-diazoesters using catalytic amounts of B(C_6_F_5_)_3_ afforded the reactive boron enolate intermediate which can be trapped by electron-rich oxygen heterocyclic compounds such as benzofuran or olefins such as indene, styrene, and alkynes to afford the cyclopropane or cyclopropene products (Scheme 16). We observed these reactions to be highly diastereoselective with the observed formation of only one diastereoisomer (yields up to 92%, 10 examples) (Scheme 16, top).^7c^

In contrast with benzofurans, indole derivatives reacted with α-aryl α-diazoesters under the same reaction conditions (CH_2_Cl_2_, 45 °C) to afford the thermodynamically controlled C–H insertion product, while benzofuran and indene afforded the kinetically controlled C2, C3 cyclopropane product. Our DFT calculations showed that for benzofurans and indenes the thermodynamic C–H insertion products are not formed as the energy barrier (32.6 kcal/mol) is too high to overcome to generate the thermodynamic C–H insertion products observed with indoles. Our DFT calculations also supported the highly diastereoselective cyclopropanated product formation. The steric hindrance in the transition state structures makes the formation of only one isomer favorable. Various olefins including 1,1-disubstituted (terminal) and 1,2-disubstituted (internal) compounds were employed for the reaction with α-aryl α-diazoesters using 10 mol % B(C_6_F_5_)_3_ and good to excellent yields (up to 83%) of the cyclopropane products were obtained (Scheme 16, middle).^7c^

At the same time, Wilkerson-Hill and co-workers also demonstrated the efficacy of B(C_6_F_5_)_3_ as a catalyst toward the cyclopropanation of olefins using α-aryl α-diazoesters. A wide substrate scope was presented to show the applicability of the methodology, and excellent yields (30 examples, up to 90%) including high diastereoselectivities (8:1 to >20:1) were reported (Scheme 16, middle).^11b^

The successful outcome from the metal-free cyclopropanation of olefins, increased our curiosity for the cyclopropenation of alkynes using catalytic triarylboranes. Our findings revealed that catalytic B(C_6_F_5_)_3_ (10 mol %) enables cyclopropenation of arylacetylenes when reacted with various α-aryl α-diazoesters (Scheme 16, bottom).^11a^ A mild reaction protocol was demonstrated to achieve a wide variety of cyclopropenated products (33 examples) in good to excellent yields (up to 85%). Interestingly, when 1-ethynyl-4-vinylbenzene bearing both terminal alkene and alkyne functionalities was employed for the reaction, only the alkene reacted with the α-aryl α-diazoester to afford the cyclopropanation product, leaving the alkyne site untouched. Furthermore, attempted syntheses of three-membered heterocyclic compounds, using the optimized reaction conditions, and employment of C=O, C=N, or C≡N bonds were not successful.

## Ring Opening Reactions

**Scheme 17:**
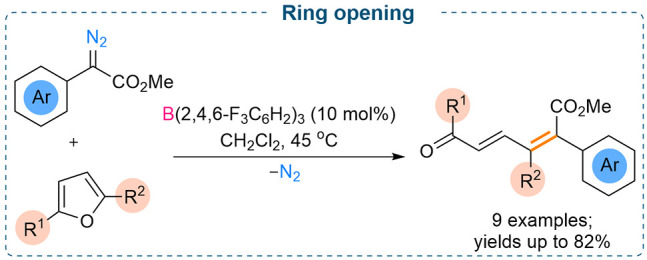
B(2,4,6-F_3_C_6_H_2_)_3_ Catalyzed Ring Opening Reactions of Furans

We have investigated the reactivities of five-membered heterocyclic compounds, such as furan, toward the α-aryl α-diazoester in the presence of catalytic amounts of B(2,4,6-F_3_C_6_H_2_)_3_ (Scheme 17).^7c^ Our investigations on the reaction between benzofuran and α-aryl α-diazoester in the presence of catalytic B(C_6_F_5_)_3_ afforded C2, C3 cyclopropanated products. However, using the same reaction conditions, with furan employed as substrate for the reaction, a ring opened product was observed. For these reactions, B(2,4,6-F_3_C_6_H_2_)_3_, a less Lewis acidic borane, was found to be catalytically more active in comparison to B(C_6_F_5_)_3_.

The yields of the ring-opened products improved significantly when comparing the unsubstituted furan to 2,5-disubstituted furan (61% to 82%). This could be attributed to the stability of the carbocation intermediate formed in the reaction. DFT studies showed that the initial O → B adduct generated is readily trapped by the furan to afford the kinetically controlled C2, C3 cyclopropane intermediate. However, the strained cyclopropanated species prefers to undergo thermal electrocyclic opening to form the resulting product.

## C–C Functionalization Reactions

**Scheme 18:**
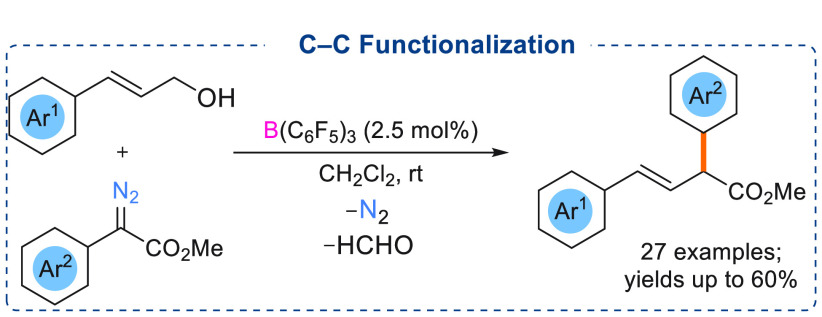
B(C_6_F_5_)_3_ Catalyzed C–C Functionalization

In 2019, Prabhu and co-workers demonstrated the use of B(C_6_F_5_)_3_ toward C–C bond functionalization of aryl allyl alcohols using donor–acceptor α-aryl α-diazoesters (Scheme 18). Reaction optimizations showed that B(C_6_F_5_)_3_ was the best catalyst for these transformations.^23^ Steric congestion around the boron center (C_6_F_5_ substituents) was found to be crucial for product formation, as similar Lewis acidic boranes such as BF_3_·OEt_2_ failed to afford the desired C–C cross-coupled products. Low catalytic loadings (2.5 mol %) with high dilution afforded the best yield of the C–C cross-coupled products, and the formation of competitive O–H insertion reaction (side products) was minimized. Various cinnamyl alcohol derivatives were reacted with different α-aryl α-diazoesters to afford C–C cross-coupled products (27 examples) in good yields (60%) (Scheme 18). Mechanistic studies revealed that Lewis acidic B(C_6_F_5_)_3_ activates the α-aryl α-diazoester to afford the carbene intermediate. Subsequent nucleophilic attack to the carbene intermediate by the allylic β-sp^2^-carbon of the allylic alcohol afforded a four-membered oxygen heterocyclic boron adduct intermediate. This eventually led to the formation of the desired C–C cross-coupled product and the borane catalyst could be regenerated. Formaldehyde was identified as the byproduct. Dimedone was used to detect the formation of formaldehyde.

## C=C Bond Forming Reactions

**Scheme 19:**
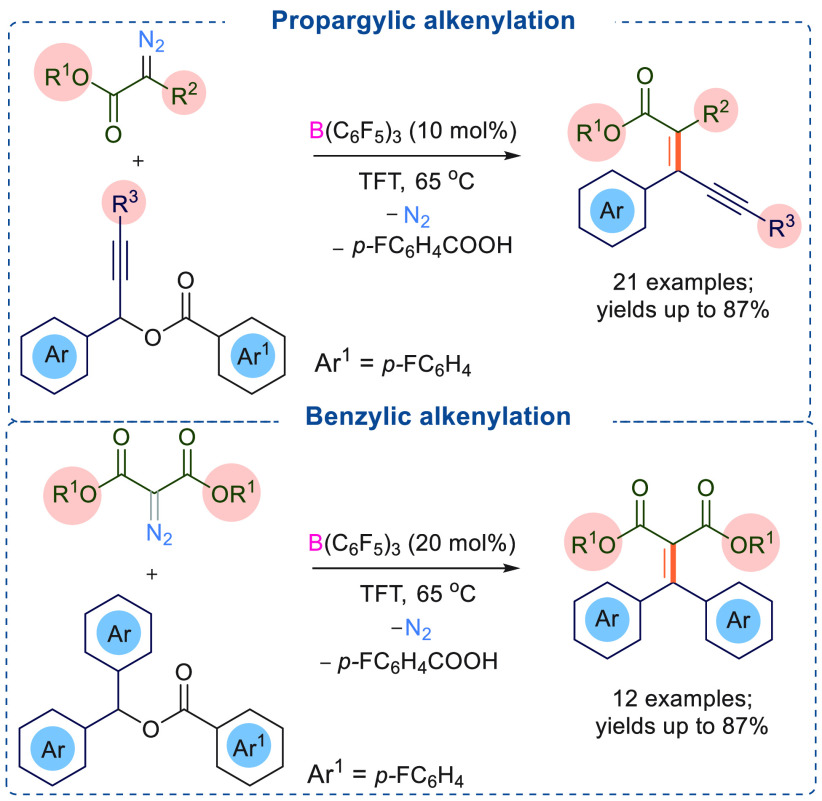
B(C_6_F_5_)_3_ Catalyzed Propargylic and Benzylic Alkenylation

Catalytic amounts of a triarylfluoroborane are also successful in C=C cross coupling reactions. In 2020, we unveiled the potential catalytic activity of Lewis acidic B(C_6_F_5_)_3_ toward an alkenylation reaction which is valuable for forming conjugated organic compounds. Using 10 mol % of B(C_6_F_5_)_3_, the decomposition of symmetrical donor–acceptor diazoesters was studied (Scheme 19).^10^

The reactive carbene intermediate generated *in situ* in the reaction mixture undergoes a reaction with aryl esters to afford C=C cross-coupled products. Alkenylation of both propargylic and benzylic sp^3^-centers were successfully demonstrated, and good to excellent yields (up to 87%) of the C=C coupled enyne products (31 examples) were obtained. The reaction mechanism was investigated using DFT studies which revealed that coordination of the borane is preferred with the carbonyl functionality of the aryl ester compared to the nitrogen/carbonyl functionality of the diazoester as observed in other studies. DFT studies showed that this is energetically favored by 3.6 kcal/mol, whereas coordination of the borane to the nitrogen center is endergonic by 13.6 kcal/mol. The O_arylester_ → B adduct formation promotes the generation of a carbenium ion.

**Figure 2:**
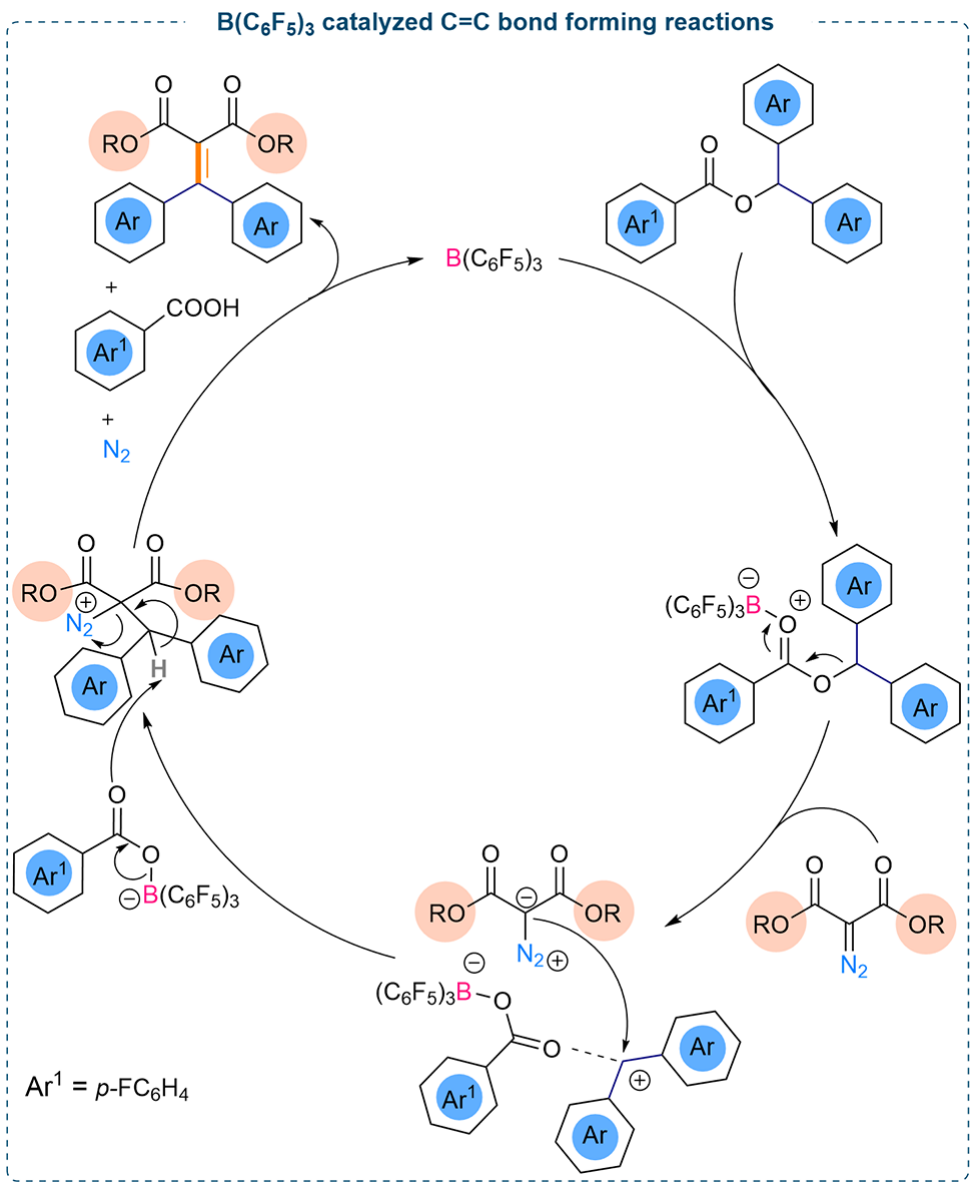
Proposed catalytic cycle for B(C_6_F_5_)_3_ catalyzed C=C bond formation.

Subsequent nucleophilic attack by the diazoester, followed by E2-type elimination of dinitrogen and 4-fluorobenzoic acid led to the formation of the C=C coupled product (Figure 2).

## Cascade Cyclizations

**Scheme 20:**
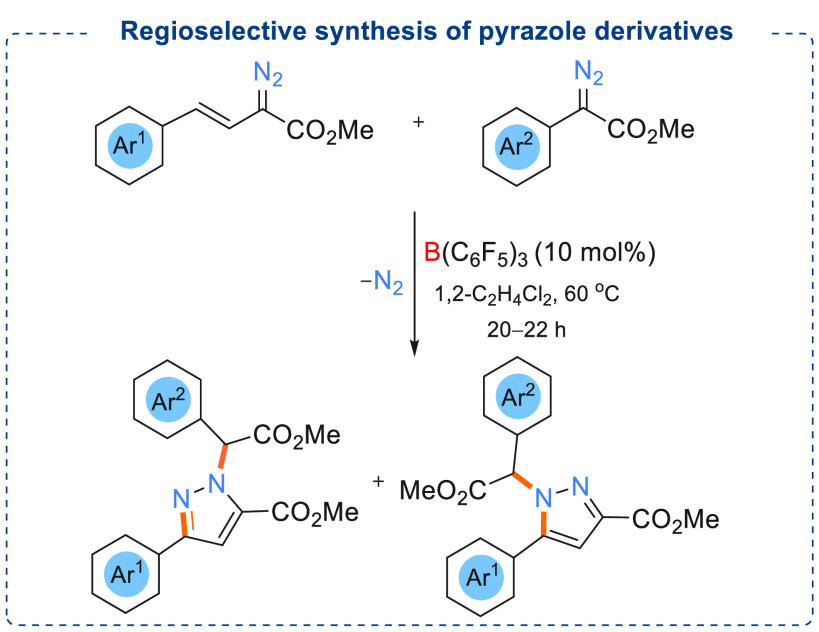
Regioselective Synthesis of Pyrazole Derivatives Using Catalytic Amounts of B(C_6_F_5_)_3_ (10 mol %)

After achieving success in the alkenylation reaction, we continued our investigation on metal-free borane catalyzed C=C cross coupling reactions. We aimed to study the stereoselective cyclization reaction using a conjugated diene^24^ system and dienophiles. With that target we performed a reaction in which vinyl diazoacetate reacted with an α-aryl α-diazoester using catalytic amounts of B(C_6_F_5_)_3_ (10 mol %) (Scheme 20).^25^ Multinuclear NMR data, high-resolution mass spectrometric data, and single crystal diffraction data confirmed the formation of a *N*-alkylated pyrazole product instead of the conjugated C=C cross-coupled or homocoupled products. Formation of the *N*-alkylated pyrazole was found to be highly regioselective. Here, selective decomposition of the α-aryl α-diazoester and subsequent reaction with the vinyl diazoacetate afforded the *N*-alkylated pyrazole products. A wide range of substrates was investigated to check the applicability of this methodology. Pleasingly good to excellent yields (36 examples, yields up to 80%) were obtained.

DFT studies disclosed that B(C_6_F_5_)_3_ activates the α-aryl α-diazoester by coordinating to the carbonyl functionality generating an electrophilic O → B adduct which is a reversible process and energetically favored by 1.5 kcal/mol. The vinyl diazo acetate readily reacts with the O → B adduct. Intramolecular cyclization (off cycle) of this intermediate led to the formation of the minor isomer through a 1,3-hydrogen shift (kinetically stable pyrazole isomer). Subsequent reaction of the boron adduct of the minor isomer with another molecule of uncoordinated minor isomer afforded the major products of these reactions.

## Summary and Outlook

In conclusion, this perspective has summarized the recent developments in triarylborane catalyzed carbene transfer reactions generated from their corresponding diazo precursors. The use of triarylboranes as alternative catalysts to transition metal catalyzed syntheses has gained popularity because of their lower toxicity and high selectivity. Strong efforts have been made to establish the mechanism by which triarylboranes interact with diazo compounds, including their mode of activation, both experimentally and theoretically. The range of organic reactions reported so far signifies their wide applicability and broad scope both stoichiometrically and catalytically toward different types of organic reactions. Extensive studies reveal that triarylboranes not only are useful as catalysts for the activation of diazo compounds to promote carbene formation but also can actively participate in the reaction to afford synthetically useful compounds. Comprehensive DFT studies have aided in our understanding of their mode of action and helps us to understand the mechanistic details. These catalysts have also been found to be useful toward stereo-controlled reactions. Focus on the development of new Lewis acidic borane catalysts, including chiral boranes, could potentially reveal the synthetic utility of main group elements toward metal-free synthesis in the future and is expected to have an impact on pharmaceutical and agrochemical relevant organic syntheses.
